# Neurodegenerative Disease Spread via the Tunneling Nanotubes

**DOI:** 10.1002/alz70855_107587

**Published:** 2025-12-27

**Authors:** Sunayana Dagar, Alexandra Fernandes, Isabella Zuniga, Chinmayee Mohapatra, Srinivasa Subramaniam

**Affiliations:** ^1^ Florida Atlantic University, Jupiter, FL, USA

## Abstract

**Background:**

Tunneling nanotubes (TNTs) are hollow tubular connections between animal cells that are membrane‐enclosed and based on F‐actin. They carry a range of biological cargo, including proteins involved in neurodegenerative diseases like Tau and mutant huntingtin (mHTT). Nonetheless, the molecular mechanisms underlying their formation, especially in the brain, remain ambiguous. Ras homolog abundant in the striatum (Rhes) is associated with Huntington's disease and tauopathy. We discovered that Rhes causes TNT‐like protrusions and facilitates the transfer of mHTT across neuronal cells in culture and the brain.

**Method:**

Using cell culture models, Co‐immunoprecipitation assays, High resolution microscopy and mass spectrometry, we investigated the role of Rhes in TNT formation and cargo transfer. Pharmacological and knockout approaches were used to assess the impact of SLC4A7 loss on TNT formation and mHTT/Tau transfer in cellular models and the intact striatum.

**Results:**

We discovered that Rhes causes TNT‐like protrusions and facilitates the transfer of mHTT across neuronal cells in culture and the brain. The preliminary findings indicated that Rhes can transmit Tau between cultivated cells. Nonetheless, the processes remain unclear. Employing an unbiased mass spectrometry technique, we identified solute carrier family four member 7 (SLC4A7), a regulator of intracellular pH, as a novel regulator of Rhes‐mediated tunneling nanotubes (TNTs). Rhes binds directly to SLC4A7 and alters intracellular pH. Pharmacological and knockout investigations indicate that the loss of SLC4A7 reduces TNTs and inhibits the dissemination of mHTT in cellular models and within the intact striatum. The preliminary findings indicate that Rhes can enhance TNT formation in microglial cultures.

**Conclusions:**

Rhes plays a crucial role in regulating the production of TNT and the dissemination of Tau and mHTT throughout the brain through neurons and microglia. Targeting Rhes presents a therapeutic possibility for Huntington's disease and tauopathies.

